# Promoting resilience among medical students using the Wadi framework: a clinical teacher’s perspective

**DOI:** 10.3389/fmed.2024.1488635

**Published:** 2024-10-18

**Authors:** Ali Ibrahim Shorbagi

**Affiliations:** Department of Clinical Sciences, College of Medicine, University of Sharjah, Sharjah, United Arab Emirates

**Keywords:** SAR framework, resilience, health professions education, test anxiety, student wellbeing

## Abstract

Resilience is essential for medical students to navigate the challenges of their education and future careers. Defined as the ability to adapt well in the face of adversity, resilience can be learned and is critical for maintaining mental health. Medical educators play a pivotal role in fostering resilience by integrating it into both formal and informal curricula, including the hidden curriculum, which can significantly influence students’ coping strategies. Research highlights the importance of resilience training in reducing depression and anxiety among students, underscoring its necessity as a core component of medical education. This essay presents the Systematic Assessment for Resilience (SAR) framework, which provides a comprehensive approach to promoting resilience through four key constructs: self-control, management, engagement, and growth. Practical strategies for each construct are discussed, including assessment mapping, time management, and the use of formative assessments to enhance students’ preparedness and self-control. Engagement is fostered through collaborative assessments, open book exams, and regular formative feedback, while growth is encouraged via self-reflection and faculty development. Implementing the SAR framework has shown positive outcomes, with students reporting reduced anxiety and improved performance. However, further exploration and institutional support are needed to fully integrate these strategies into medical education. The SAR framework offers a feasible and effective method for cultivating resilience, contributing to students’ mental well-being and equipping them to face future challenges in their medical careers. Continued refinement and broader institutional adoption will be crucial to sustaining the impact of resilience training in medical education.

## Introduction

Resilience is defined as the process of adapting well in the face of adversity, trauma, tragedy, threats, or even significant sources of stress (1), and has been described in health professions education as the ability to cope with adversity that can be learned (2). Recent studies suggest that medical educators have a unique role in fostering resilience by integrating it into both formal and informal curricula (3). Furthermore, the hidden curriculum, which includes the unspoken or implicit messages conveyed through the learning environment, can significantly influence students’ resilience (4). Integrating resilience training into the medical curriculum can help students develop the necessary coping strategies to navigate the pressures of medical education (5).

The role of resilience in managing mental health among medical students is particularly important, as highlighted by cross-sectional studies showing that resilient students report lower levels of depression and anxiety (6). These findings underscore the need for resilience training to be a core component of medical education programs.

**Figure 1:**
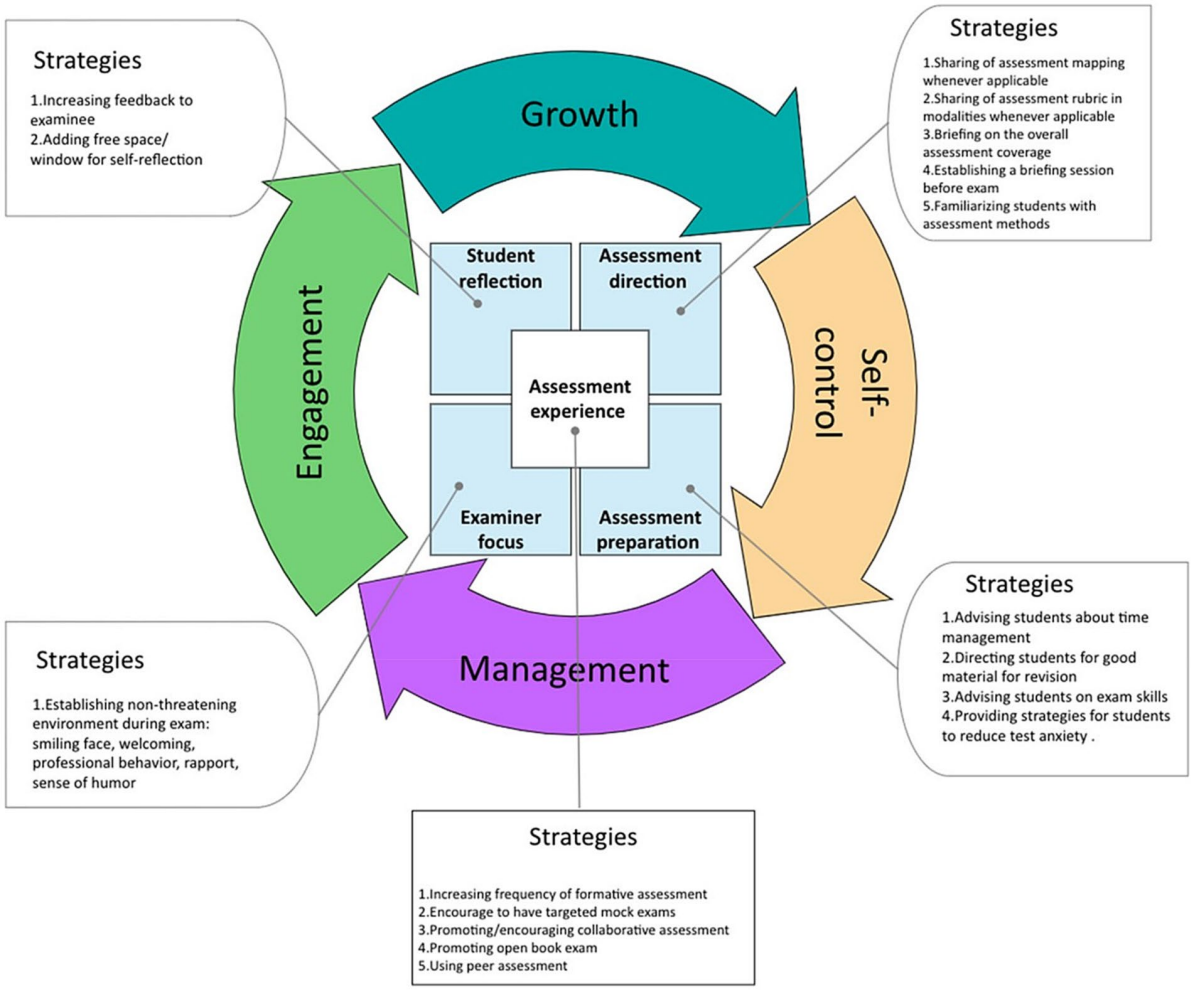
The framework of systematic assessment for resilience (SAR) [Adopted from Wadi et al. (7)].

As an educator deeply invested in the holistic development of medical students, I recognize the immense pressures they face and the critical need for resilience in navigating these challenges. The Systematic Assessment for Resilience (SAR) framework developed by Wadi et al. (7) offers a comprehensive approach to fostering resilience by enhancing self-control, management, engagement, and growth among students (Figure 1). A practical guide to using the SAR framework is depicted in Table 1. This essay outlines my perspective on promoting resilience using the SAR framework, drawing from practical strategies I have implemented and insights gained from the framework’s application (8).

**Table 1:** Using systemic assessment to promote resilience (SAR): a practical guide.

Assessment phase		Resilience strategies	Example	Methods of sharing/distribution
Pre - course	Assessment direction	Sharing of assessment mapping/blueprinting whenever applicable	General blueprint	Course booklet Electronic platform (e.g., LMS)
		Sharing of assessment rubric in modalities whenever applicable	Rubric	Course booklet Electronic platform (e.g., LMS)
		Briefing on the overall assessment coverage	Overall assessment briefing	Course booklet Electronic platform (e.g., LMS)
		Establishing a briefing session before exam	Before exam briefing	Class session Electronic platform (e.g., LMS)
		Familiarizing students with assessment methods	Familiarizing assessment methods	Class session Electronic platform (e.g., LMS)
	Assessment preparation	Advising students about time management and study skills	Links to relevant materials (YouTube, Websites … etc) Discussion or webinar https://youtu.be/MR7e1kPp_ys	Electronic platform (e.g., LMS) Text advice through WhatsApp
		Directing students for good material for revision	Good handouts for revision Concise reference books	• Electronic platform (e.g., LMS)
		Advising students on exam skills	Links to relevant materials (YouTube, Websites … etc) Discussion or webinar	Electronic platform (e.g., LMS) Text advice through WhatsApp
		Providing strategies for students to reduce test anxiety	Advice about personal/selfcare (nutrition, sport, sleep) Advice about relaxation techniques (deep breathing) Discussion about these strategies https://youtu.be/4PgEIlewf7Y	Electronic platform (e.g., LMS) Text advice through WhatsApp
During - course	Assessment experience	Increasing frequency of formative assessment	More formative assessment	
		Encourage to have targeted mock exams	Mock exam	
		Promoting/encouraging collaborative assessment	Collaborative assessment https://youtu.be/RNOC2OHP6Vs	Class session Electronic platform (e.g., LMS)
		Promoting open book exam	Open book exam https://youtu.be/-xVwAdCgdlQ	Class session Electronic platform (e.g., LMS)
		Using peer assessment	Peer assessment https://youtu.be/2hRu5i-gfXo	Class session Electronic platform (e.g., LMS)
		Introducing progress testing	Progress test https://youtu.be/Mc_-fVmEf1M	Class session Electronic platform (e.g., LMS)
On exam day	Examiner focus	Establishing non-threatening environment during exam	Smiling face Welcoming Professional behavior Rapport Sense of humor	On the exam day
	Student reflection	Increasing feedback to examinee	https://youtu.be/s8Jl-8JLxdo	On the exam day
		Sharing the key answer if applicable		On the exam day
		Adding free space/window for self-reflection	https://youtu.be/SntBj0FIApw	On the exam day

### Self-control: empowering students to govern themselves

The first construct of the SAR framework, self-control, emphasizes students’ ability to govern themselves and face adversity. To promote self-control, it is essential to create an environment where students feel supported and empowered. Here are some strategies:

1. **Assessment Mapping and Blueprinting:** Sharing detailed assessment maps and blueprints helps students understand the scope and expectations of their evaluations. As the coordinator of the Internal Medicine clerkship, I have ensured that blueprints for written exams and rubrics for clinical assessments are readily available on the Learning Management System (LMS), Blackboard Ultra. This guides students to focus on core topics and prepare effectively. Using course booklets and electronic platforms, I have made assessment rubrics available and directed students to these resources during orientation sessions.
2. **Time Management and Study Skills:** Providing resources on time management and effective study techniques is crucial. I have shared links to relevant YouTube videos and conducted academic advising sessions to support students with low performance, helping them develop better study habits and manage their time efficiently. Platforms like WhatsApp have been useful for sharing text advice and resources. I have also conducted webinars on time management and study skills, with links shared via LMS and emails.
3. **Reducing Test Anxiety:** Addressing test anxiety through discussions on personal self-care, relaxation techniques, and exam skills can significantly enhance self-control. While I have yet to implement formal sessions on these topics, I plan to create videos and conduct webinars to guide students on reducing test anxiety. Sharing advice about nutrition, sports, sleep, and relaxation techniques via electronic platforms will be part of this strategy.

### Management: utilizing resources effectively

Effective management of available resources is vital for overcoming obstacles. The SAR framework encourages students to leverage these resources efficiently:

1. **Familiarizing Students with Assessment Methods:** Conducting on-campus workshops and formative assessments helps students become familiar with different evaluation methods. I have organized workshops on procedural skills and case-based discussions, simulating the format of summative assessments and providing formal feedback to enhance student preparedness. Using electronic platforms, I have shared formative assessment methods and resources to aid in student understanding.
2. **Providing Good Study Materials:** Sharing concise reference materials and recommended textbooks on the LMS ensures students have access to high-quality revision resources. Highlighting these resources during orientation sessions further directs students to reliable study materials. Lists of good handouts for revision and links to concise reference books have been made available to students electronically.
3. **Mock Exams and Formative Assessments:** Increasing the frequency of formative assessments and organizing mock exams can significantly boost students’ confidence and performance. While I have incorporated formative assessments, I aim to introduce targeted mock exams to provide more comprehensive preparation. Promoting open book exams and collaborative assessments through class sessions and electronic platforms are future goals to enhance the learning experience.

### Engagement: commitment and perseverance

Engagement, the third construct, focuses on students’ commitment to pursuing challenges with perseverance. Strategies to enhance engagement include:

1. **Collaborative and Peer Assessment:** Encouraging collaborative assessments and peer feedback can foster a supportive learning environment. Although I have limited experience with these methods, I recognize their potential and plan to explore their applicability in future assessments. I will use on-campus sessions and online resources to implement peer assessment practices.
2. **Promoting Open Book Exams:** While not currently part of our undergraduate assessment strategies, introducing open-book exams for certain assignments can encourage deeper learning and resourcefulness among students. Sharing resources and guidelines for open book exams on platforms like Blackboard will be an essential step.
3. **Formative Feedback:** Providing regular, constructive feedback is essential for continuous improvement. I have emphasized formal feedback in formative assessments, and I aim to incorporate more opportunities for students to receive and reflect on feedback. Encouraging weekly formative assessments with formal feedback in hospitals is part of this strategy.

### Growth: ongoing development

The final construct, growth, focuses on students’ continuous development to face future challenges. Promoting growth involves:

1. **Student Reflection:** Encouraging self-reflection through written reflections and surveys helps students evaluate their performance and identify areas for improvement. Incorporating reflection within e-portfolio submissions has been a step in this direction, but I plan to create more structured reflection sessions. Adding free space for self-reflection during assessments can also aid in this process.
2. **Faculty Development:** Training faculty members to create a non-threatening exam environment and provide effective feedback can significantly impact students’ growth. Conducting faculty development meetings on good clinical teaching practices has been beneficial, and I will continue to emphasize the importance of fostering a supportive academic culture. Encouraging examiners to establish a welcoming and professional behavior during exams is crucial

### Implementing the SAR framework: practical insights

Based on my experience with the SAR framework, several key insights have emerged:

1. **Feasibility and Practicality:** The SAR guidelines are practical and can be seamlessly integrated into daily educational practices. The framework’s checklists and resources make it easy for educators to implement resilience-promoting strategies.
2. **Positive Student Feedback:** Students have responded positively to the SAR framework, reporting reduced anxiety, improved performance, and greater enjoyment of their rotations. These outcomes highlight the framework’s effectiveness in enhancing resilience and overall well-being.
3. **Challenges and Future Directions:** While the SAR framework has shown promise, certain strategies, such as collaborative assessments and progress testing, require further exploration and institutional support. It is crucial to engage curriculum committees and seek official approval for broader implementation.

## Discussion

The SAR framework has demonstrated promising results in fostering resilience among medical students. However, it is essential to consider the broader context in which resilience is cultivated. Integrating resilience training into the medical education continuum is crucial for supporting students’ mental health and well-being (1). Medical educators must critically reflect on how their conceptualizations of resilience influence their approach to teaching and supporting learners.

The literature suggests that resilience is not solely an individual trait but a dynamic process influenced by the learning environment, social support, and institutional culture (2). Studies show that medical students who develop resilience through structured programs are better equipped to manage stress and avoid burnout, which is critical for maintaining mental health throughout their education and careers (6). By fostering a culture of resilience through formal, informal, and hidden curricula, medical schools can better support students in developing the skills needed to manage stress and thrive in their careers (5).

While the SAR framework provides a comprehensive approach to fostering resilience in medical students and demonstrates practical applications in a tutor-led environment, it is equally critical to extend the discussion to include the clinical setting, where students face significant emotional and psychological challenges. Clinical placements offer unique and essential opportunities for developing resilience by allowing students to apply theoretical concepts to real-life patient care, manage uncertainty, and cope with the pressures of medical practice (7).

### Resilience in the clinical environment

Clinical environments are inherently unpredictable, presenting medical students with emotionally charged situations such as difficult patient interactions, high-stakes decision-making, and exposure to suffering and death. These settings require students to develop coping mechanisms that enable them to adapt to high-pressure situations and maintain emotional equilibrium. Research supports the idea that students who develop resilience in these environments are better prepared to manage stress and avoid burnout (6). For example, a study by Bird et al. (2) found that medical students who participated in resilience training during clinical rotations reported reduced anxiety, greater emotional regulation, and improved coping strategies for handling difficult patient interactions.

Resilience in clinical settings is often fostered through a combination of exposure to challenging experiences and structured reflective practice. By navigating the complexities of patient care, students learn to tolerate uncertainty and develop emotional regulation skills. Research highlights that structured debriefing sessions during clinical rotations help students process these experiences and develop the emotional fortitude necessary for future medical practice (5). Reflective practices, such as guided reflection and journaling, have been shown to enhance students’ ability to understand their emotional responses, ultimately contributing to improved resilience (9).

### Mentorship and peer support

Mentorship from experienced clinicians is another crucial component in developing resilience in clinical settings. Mentors provide not only clinical guidance but also emotional support, helping students navigate difficult situations. Mentors can model how to cope with the pressures of clinical practice, such as managing patient loss, balancing personal and professional demands, and maintaining mental well-being in a demanding environment (3). In one study, students who engaged in mentor-guided reflective sessions reported higher levels of emotional resilience and improved coping mechanisms (5). Structured mentorship programs, where students are encouraged to reflect on their experiences and discuss strategies for emotional resilience, can significantly enhance their ability to cope with stress (9).

Peer support also plays a vital role in resilience-building. Students benefit from discussing their clinical experiences with peers, sharing coping strategies, and providing mutual support. Peer reflection groups, where students can debrief and process their emotions together, foster a supportive community that is crucial for resilience-building. Research indicates that students who participate in regular peer reflection sessions are more likely to develop positive coping strategies and resilience in the face of adversity (6).

### Practical strategies for resilience training in clinical settings

While the SAR framework offers a robust structure for resilience-building in theoretical settings, it is equally applicable to clinical contexts. Practical strategies for integrating the SAR framework into clinical rotations include:

1. **Resilience Workshops**: Implementing workshops at the beginning of clinical placements that focus on stress management, emotional regulation, and coping strategies for high-pressure situations. These workshops can equip students with the tools they need to navigate the challenges of clinical practice, such as managing uncertainty, patient suffering, and complex decision-making. Research has shown that students who attend resilience workshops during clinical rotations experience reduced burnout and increased emotional regulation (2).
2. **Reflective Debriefing Sessions**: Structured debriefing sessions after particularly challenging clinical encounters, such as dealing with patient deaths or difficult diagnoses, provide students with the opportunity to reflect on their emotional responses. These sessions help students process their experiences, develop emotional regulation skills, and prepare for future challenges (5). Studies have shown that such reflective practices contribute to improved emotional resilience and reduced levels of stress and anxiety (3).
3. **Formative Feedback on Emotional Resilience**: Feedback during clinical rotations should extend beyond clinical competence to include emotional and psychological well-being. Supervisors can provide formative feedback on how students handle stress, interact with patients, and manage emotionally challenging situations. This feedback helps students develop self-awareness and emotional resilience, essential components of professional development in healthcare (9).
4. **Peer Support Systems**: Incorporating peer support structures within clinical rotations allows students to share experiences and coping strategies. Peer reflection groups or regular check-ins where students discuss their clinical encounters provide a sense of community and shared understanding, which is critical for resilience (6).

### The importance of institutional support

While the SAR framework and associated strategies can help develop individual resilience, institutional support is critical for sustaining these efforts. Medical schools should prioritize resilience training by integrating it into the curriculum and ensuring that students have access to mental health resources, counseling, and structured mentorship programs (9). Institutional culture plays a significant role in fostering resilience, as students who feel supported by their academic and clinical environments are more likely to thrive. Promoting a culture of openness, where students are encouraged to discuss their emotional challenges, can also reduce the stigma associated with stress and mental health issues in the medical profession (2).

## Conclusion

The SAR framework offers a structured approach to promoting resilience in medical students, focusing on self-control, management, engagement, and growth. While it has shown success in theoretical settings, its application to clinical environments is essential for fully preparing students for the demands of medical practice. By incorporating resilience training into clinical rotations—through mentorship, peer support, and reflective practices—medical educators can help students develop the coping mechanisms needed to navigate the complexities of patient care. Institutional support is equally important in creating a culture that prioritizes mental well-being and resilience, ultimately ensuring that students are prepared for the emotional and psychological challenges of their future careers in medicine.

## Data Availability

The original contributions presented in the study are included in the article/supplementary material, further inquiries can be directed to the corresponding author.
